# The Pattern of Retinal Nerve Fiber Layer and Macular Ganglion Cell-Inner Plexiform Layer Thickness Changes in Glaucoma

**DOI:** 10.1155/2017/6078365

**Published:** 2017-08-13

**Authors:** Jin A Choi, Hye-Young Shin, Hae-Young Lopilly Park, Chan Kee Park

**Affiliations:** ^1^Department of Ophthalmology, College of Medicine, St. Vincent's Hospital, Catholic University of Korea, Suwon, Republic of Korea; ^2^Department of Ophthalmology, College of Medicine, Uijeongbu St. Mary's Hospital, Catholic University of Korea, Uijeongbu, Republic of Korea; ^3^Department of Ophthalmology, College of Medicine, Seoul St. Mary's Hospital, Catholic University of Korea, Seoul, Republic of Korea

## Abstract

**Background/Aims:**

To investigate the patterns of retinal ganglion cell damage at different stages of glaucoma, using the circumpapillary retinal nerve fiber layer (RNFL) and macula ganglion cell-inner plexiform layer (GCIPL) thicknesses.

**Methods:**

In 296 eyes of 296 glaucoma patients and 55 eyes of 55 healthy controls, the correlations of mean deviation (MD) with the superior and inferior quadrant RNFL/GCIPL thickness (defined as the average of three superior and inferior sectors, resp.) were analyzed.

**Results:**

In early to moderate glaucoma, most of the RNFL/GCIPL thicknesses had significant positive correlations with the MD. In advanced glaucoma, the superior GCIPL thickness showed the highest correlation with MD (*r* = 0.495), followed by the superior RNFL (*r* = 0.452) (all; *P* < 0.05). The correlation coefficient of the inferior RNFL thickness with MD (*r* < 0.471) was significantly stronger in early to moderate glaucoma compared to that in advanced glaucoma (*r* = 0.192; *P* < 0.001). In contrast, the correlations of the superior GCIPL thickness with MD (*r* = 0.452) in advanced glaucoma was significantly stronger compared to that in early to moderate glaucoma (*r* = 0.159; *P* < 0.001).

**Conclusions:**

The most preserved region in advanced glaucoma appears to be the superior macular GCIPL, whereas the most vulnerable region for initial glaucoma is the inferior RNFL around the optic disc.

## 1. Introduction

Glaucomatous damage usually spares the horizontal meridian in the early stage and occurs asymmetrically across the horizontal meridian [1]. In the early stages of glaucoma, the inferior retina, corresponding to superior hemifield, is involved more frequently [2, 3], with faster progression, compared with the superior retina, corresponding to the inferior hemifield [4]. In terms of progression, the retinal nerve fiber layer (RNFL) on the inferotemporal side (324–336°) is the most common location of progressive changes detected by optical coherence tomography (OCT) [5]. Studies of the disparity in glaucomatous damage between the superior and inferior retina have focused on the relatively early stages of glaucoma [4–6]. By contrast, in advanced glaucoma, few studies have addressed the pattern of structural loss because, in this stage, extensive RNFL loss in both the superior and inferior retina has already occurred, and the assessment of the severity of disease is based largely on visual field (VF) parameters.

It has been well known that the selective retinal ganglion cell loss is a pathological hallmark of the glaucoma optic neuropathy. That begins at optic disc lamina as it is compressed and deformed by intraocular pressure (IOP) and makes the axonal damage as a consequence [7]. In practical field, we can observe them as a cupping enlargement in the disc and an RNFL defect. The next sequence might be a soma change, and that would be a natural history of the retinal ganglion cell loss in the glaucomatous optic neuropathy [8–10]. But unfortunately, this sequence is hardly recognizable by the clinical observations.

Cirrus high-definition- (HD-) OCT (Carl Zeiss Meditec, Dublin, CA), a commercial OCT device, can measure the macular ganglion cell-inner plexiform layer (GCIPL) thickness using a ganglion cell analysis (GCA) algorithm [11–13]. With an aid of this recent advancement of OCT technology, we can now figure out the structure of the whole retinal ganglion cell from the dendrite/soma (GCIPL) to the axon (cpRNFL). So we hope, in this study, we can evaluate the whole sequence of the retinal ganglion cell damage by observing the cpRNFL and GCIPL thickness changes in different stages of glaucoma from the beginning to the end. This would help us clinically to know the sensitive sites and the parameters for glaucoma severity in different stages and, in addition, help us academically to understand the natural history of the retinal ganglion cell death in glaucoma.

## 2. Patients and Methods

### 2.1. Study Samples

The medical records of all consecutive patients with open-angle glaucoma and healthy controls examined by a glaucoma specialist (CKP) between April 2012 and May 2013 at the glaucoma clinic of Seoul St. Mary's Hospital (Seoul, Korea) were reviewed retrospectively. This study was performed according to the tenets of the Declaration of Helsinki after approval by our institutional review board. When both eyes met the inclusion criteria, one eye was chosen randomly for the study.

All subjects underwent a medical history review, measurement of the best-corrected visual acuity, refraction, slit-lamp biomicroscopy, gonioscopy, Goldmann applanation tonometry, dilated stereoscopic examination of the optic disc, disc and red-free fundus photography (Canon, Tokyo, Japan), standard perimetry (24-2 Swedish Interactive Threshold Algorithm SAP, Humphrey Field Analyzer II; Carl Zeiss Meditec, Dublin, CA), and spectral-domain OCT (Cirrus HD-OCT; Carl Zeiss Meditec). All included subjects had a best-corrected visual acuity ≥ 20/40, spherical refraction within ± 6.0 diopters, cylinder correction within ± 3.0 diopters, and normal and open anterior chamber angle by gonioscopy, and no history or evidence of retinal disease or nonglaucomatous optic nerve diseases. Patients with neurological or intraocular diseases that could cause VF defects and eyes with consistently unreliable VF results (defined as >25% false-negative results, >25% false-positive results, or >20% fixation losses) were excluded from the study.

Glaucoma was diagnosed when patient had a glaucomatous VF defect on two consecutive, reliable VF examinations and by the presence of typical glaucomatous optic disc damage (diffuse or localized rim thinning on stereoscopic color fundus photographs), irrespective of the level of IOP. A glaucomatous VF change was defined as the consistent presence of a cluster of three or more points on the pattern deviation plot with a probability of occurrence of <5% in the normal population, having one point with a probability of occurrence in <1% of the normal population, glaucoma hemifield test results outside the normal limits, or a pattern standard deviation (PSD) with *P* < 5%.

The severity of the glaucomatous damage was classified into early, moderate, and advanced stages according to the Hodapp-Parrish-Anderson criteria [14]. Healthy control eyes had an IOP ≤ 21 mmHg, no glaucomatous disc appearance, no visible RNFL defect on red-free RNFL photography, and a reliable normal VF test (an MD or PSD within the 95% confidence interval (CI) and a normal glaucoma hemifield test).

### 2.2. OCT Imaging

All subjects underwent imaging using spectral-domain OCT (Cirrus high-definition-OCT; Carl Zeiss Meditec) to acquire one optic disc (Optic Disc Cube 200 × 200 protocol) scan and one macular (Macular Cube 514 × 128 protocol) by the same operator on the same day. The circumpapillary scan allowed measurement of the RNFL thickness, whereas the macular scan allowed determination of the macular GCIPL thickness using the GCA algorithm [11–13, 15]. The circumpapillary scan measures on the 6 mm × 6 mm data and the GCA algorithm detect and measure macular GCIPL thickness within an annulus with inner vertical and horizontal diameters of 1 and 1.2 mm, respectively, and outer vertical and horizontal diameters of 4 and 4.8 mm, respectively. Image quality was assessed by an experienced examiner blinded to the patient's identity and other test results. Only well-focused, well-centered images without eye movement with signal strengths of 6/10 or greater were used.

For the cpRNFL thickness measurements, the average, superior, and inferior quadrant and clock-hour thicknesses were used. The following GCIPL thickness measurements were analyzed: average, sectoral (superior (S), superonasal (SN), inferonasal (IN), inferior (I), inferotemporal (IT), superotemporal (ST)), the superior GCIPL thickness (defined as the average of the measurements in the S, SN, and ST sectors), and the inferior GCIPL thickness (defined as the average of the measurements in the I, IN, and IT sectors). Right eye orientation was used for the documentation of measurements in left eye.

### 2.3. Cross-Sectional Analysis of VF Progression Patterns in Advanced Stage Glaucoma

To visualize the average maps at different disease stage in advanced glaucoma, eyes with advanced stage glaucoma were further classified into 5 subgroups according to MD (I: −12 dB ≥ MD > −15 dB, II: MD > −18 dB, III: MD > −21 dB, IV: MD > −24 dB, V: MD ≤ −24 dB). Within each subgroup, threshold sensitivity map numeric values of the 24-2 VF tests were averaged for each VF test point, generating 1 average map for each subgroup. Based on the maximum and minimum threshold sensitivity values of all average maps, a linear grayscale was generated and applied to all maps [16].

### 2.4. Data Analysis

Multiple comparisons among the groups were conducted using one-way analysis of variance (ANOVA) and Tukey's test. The independent Student's *t*-test was used to compare the superior and inferior cpRNFL/GCIPL thicknesses in each group. The associations of MD and cpRNFL/GCIPL thicknesses were evaluated using Pearson's correlation coefficient. To compare the associations between the VF MD and sectoral OCT measurements obtained using ONH scan and macular GCIPL modes, the bootstrap method (1000 replicates) using the *R* means from 1000 samples reported for each relationship was used to assess the significance of differences between any two correlation coefficients. And a *t*-test was done to test the null hypothesis that the *R* value between two models is equal. To evaluate the goodness of fit of the prediction curve to our dataset, we plotted locally weighted scatterplot smoothing (LOWESS) curves. All statistical analyses were performed using SPSS software ver. 17.0 (SPSS, Chicago, IL). Values of *P* < 0.05 (two-tailed) were considered to be significant.

## 3. Results

The study included 296 eyes of 296 patients with open-angle glaucoma and 55 eyes of 55 healthy controls. According to the glaucoma classification criteria, 190, 58, and 48 eyes had early, moderate, and advanced glaucoma, respectively.

Among the demographic and ocular parameters, age and central corneal thickness (CCT) differed significantly between the healthy control and glaucomatous eyes (*P* < 0.001 and 0.004, resp.) (Table 1).

**Figure 1:**
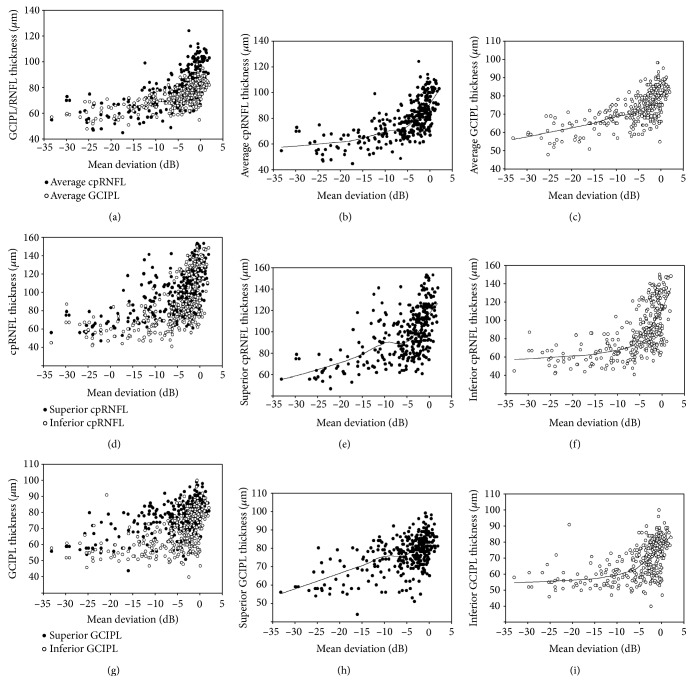
Scatterplot showing the average, superior, and inferior cpRNFL/GCIPL thicknesses according to glaucoma severity assessed using mean deviation (MD) in the study population.

Figure 1 shows the overall patterns of the average, superior, and inferior cpRNFL/GCIPL thicknesses according to disease severity assessed by the MD in the study population. The LOWESS plot suggests a curvilinear relationship of the MD with the average, superior, and inferior cpRNFL/GCIPL thicknesses. In advanced disease, the relationship between the MD and the superior versus inferior cpRNFL/GCIPL thicknesses differed. The relationship with the MD was stronger for the superior cpRNFL/GCIPL (Figures 1(e) and 1(h)) than that for the inferior cpRNFL/GCIPL thicknesses (Figures 1(f) and 1(i)). Particularly, the superior GCIPL thickness had a strong relationship with the MD in advanced disease (Figure 1(h)).

**Figure 2:**
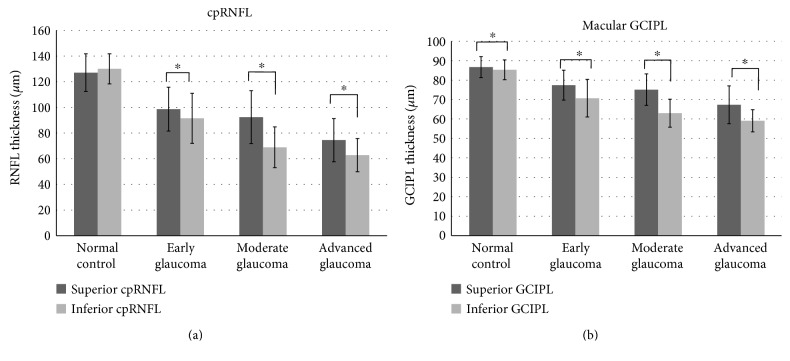
Comparisons of the superior and inferior cpRNFL/GCIPL thickness in healthy controls, in early, moderate, and advanced glaucomatous eyes. ^∗^*P* < 0.05.

In the healthy controls, the inferior cpRNFL (130.07 ± 11.6 *μ*m) tended to be thicker than the superior cpRNFL (126.96 ± 14.6 *μ*m), but with marginal significance (*P* = 0.079; Figure 2(a)), whereas the superior GCIPL (86.71 ± 4.53 *μ*m) was significantly thicker than the inferior GCIPL (85.25 ± 4.40 *μ*m; *P* < 0.001; Figure 2(b)). In early, moderate, and advanced glaucoma, the inferior cpRNFL/GCIPL thicknesses were persistently and significantly thinner than the superior cpRNFL/GCIPL thicknesses (all *P* < 0.05; Figures 2(a) and 2(b)).

In early to moderate glaucoma, most of the cpRNFL/GCIPL thicknesses had significant positive correlations with the MD; particularly, the average (*r* = 0.477) and inferior cpRNFL (*r* = 0.471) thickness showed relatively high correlations with the MD (Table 2). In advanced glaucoma, the superior GCIPL thickness (*r* = 0.495) was most strongly correlated with the MD, followed by the superior cpRNFL thickness (*r* = 0.452) and inferior GCIPL (*r* = 0.342) thickness (all *P* < 0.05). The correlation coefficient of the inferior RNFL thickness with MD (*r* = 0.471) was significantly stronger in early to moderate glaucoma compared to that in advanced glaucoma (*r* = 0.192; *P* < 0.001). In contrast, in advanced glaucoma, the correlations of the superior GCIPL thickness with MD (*r* = 0.495) were significantly stronger compared to those in early to moderate glaucoma (*r* = 0.159; *P* < 0.001).

**Figure 3:**
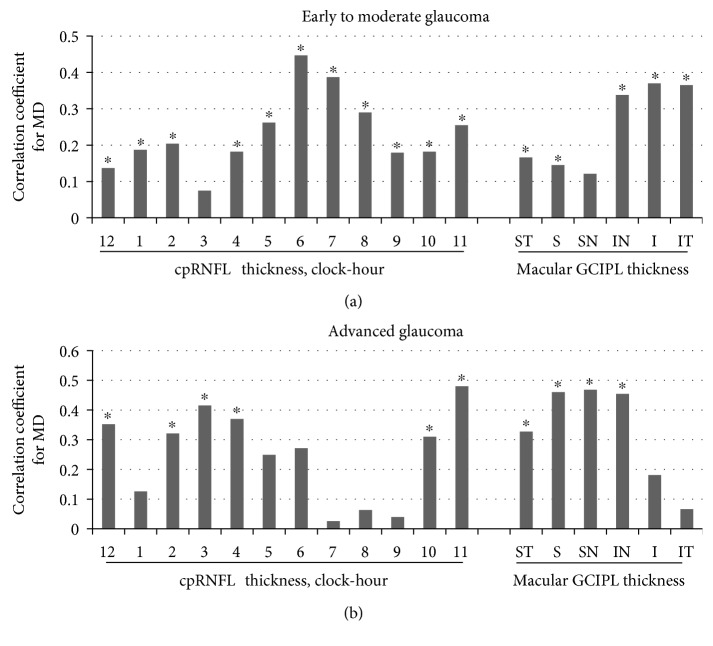
Correlation coefficients for the MD from the Humphrey visual field analysis with the cpRNFL and GCIPL sectoral parameters by OCT in glaucomatous eyes at different stages. ^∗^*P* < 0.05.

Figure 3 shows the correlation coefficients of the MD with the cpRNFL and GCIPL sectoral parameters in early to moderate and advanced glaucoma. In early to moderate glaucoma, the inferior cpRNFL clock-hour thickness (clock hours 6–8) and inferior GCIPL sectors (IN, I, and IT) showed relatively high correlations with the MD. However, in advanced glaucoma, the superior cpRNFL clock-hour thicknesses (clock hours 10–12) and superior GCIPL sectors (ST, S, and IT) and IN sector showed relatively high correlations with the MD.

**Figure 4:**
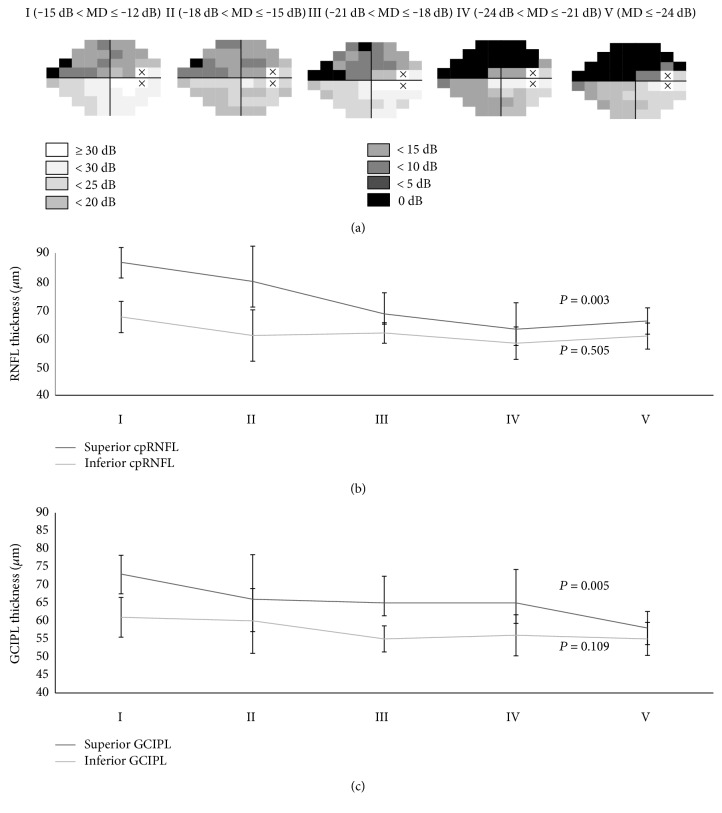
Average threshold sensitivity maps of the advance glaucoma subgroups (I to V) in cross-sectional analysis. The grayscale applied is shown on the top left. With increasing disease severity, the deepening and enlargement of scotomas occurred more frequently in superior hemifield. The scotomas in both hemifields spread toward the physiologic blind spot and toward the nasal periphery in an arcuate pattern. The scotomas in superior hemifield occurred very closely to the area corresponding to the papillomacular bundle, whereas the parafoveal area of inferior hemifield was relatively spared (a). With the disease severity (I to V), superior RNFL and superior GCIPL thickness became significantly thinner (*P* = 0.003 and 0.005, resp.), whereas changes of inferior RNFL and inferior GCIPL were not significant (*P* = 0.505 and 0.109, resp.) (b and c). “X” indicates blind spot; dB = decibel.

Figure 4(a) shows the average threshold sensitivity maps of the 24-2 VF tests for subgroups by disease severity assessed by MD in advanced glaucoma. With increasing disease severity, the deepening and enlargement of scotomas occurred more frequently in superior hemifield. The scotomas in both hemifields spread toward the physiologic blind spot and toward the nasal periphery in an arcuate pattern. The scotomas in superior hemifield occurred very closely to the area corresponding to the papillomacular bundle, whereas the parafoveal area of inferior hemifield was relatively spared. With the disease severity, superior cpRNFL thickness became significantly thinner (*P* = 0.003) with disease severity, whereas changes of inferior cpRNFL was not significant (*P* = 0.505; Figure 4(b)). Superior GCIPL thickness became significantly thinner (*P* = 0.005) with disease severity, whereas changes of inferior GCIPL was not significant (*P* = 0.109; Figure 4(c)).

## 4. Discussion

Our study was designed with the purpose of investigating the patterns of retinal ganglion cell damage at different stages of glaucoma, using the cpRNFL and macula GCIPL thicknesses. We observed that the inferior cpRNFL change had the highest correlation with the MD in early stage glaucoma as well as the superior GCIPL change in advanced glaucoma (Table 2).

Through the patterns of structural loss observed in the present study, the natural history of retinal ganglion cell degeneration may be inferred. At initial stage, the glaucomatous damage remains localized involving the inferior (mainly) or superior temporal RNFL. With the progression of disease, lesions are expanded and deepened and new lesion is developed [5, 17]. Finally, at the far advanced stage, extensive glaucomatous damage occurs, with the relative preservation of retinal ganglion cell bodies at superior macula area. This is further supported by the finding that the VF in far advanced glaucoma is gradually reduced to small central VF area, called the “central isle,” which involves mainly the inferior temporal parafoveal VF area next to the blind scotoma [18]. In accordance with this, we found that superior cpRNFL/GCIPL thickness corresponding to inferior hemifield became significantly thinner, whereas changes of inferior cpRNFL/GCIPL corresponding to superior hemifield were not significant (Figures 4(b) and 4(c)). It is well known that OCT is less clinically useful due to a “floor effect” of RNFL thickness. At this stage of disease, sequential VF tests are more reliable to detect progression. Our study results suggest that the detection of progressive thinning of the superior cpRNFL/GCIPL may be useful in determining the progression of the disease in advanced glaucomatous eyes before reaching a “floor effect.”

The reason for the relative sparing of the retinal ganglion cells in the superior macula at advanced stage remains obscure. First, the inferior temporal lamina cribrosa has larger single pores and the least supporting connective tissue [3, 19], which might render the region more susceptible to glaucomatous damage. In addition, the superior GCIPL is reported to be thicker than the inferior GCIPL in normal populations [20]. In this study, the superior GCIPL was also significantly thicker on average than the inferior GCIPL in healthy controls (Figure 2(b)). These findings suggest that relatively more ganglion cells exist in the superior macula compared to the inferior macula.

In glaucoma, structural loss precedes functional changes [21, 22]. Accordingly, most of the RNFL/GCIPL thicknesses, particularly the average and inferior cpRNFL/GCIPL thicknesses, had significant correlations with disease severity assessed by the MD in early to moderate glaucoma (Table 2 and Figure 3()). This observation is in agreement with previous reports that the RNFL loss was most evident at the inferotemporal meridian in the frequency distribution and progression analysis [5, 22]. The inferior quadrant RNFL has also been considered to be the best RNFL parameters discriminating glaucoma from normal control [11, 23]. Regarding the GCIPL parameters, the inferotemporal GCIPL sectors also showed the high diagnostic accuracy in early glaucoma, regardless of the initial location (superior versus inferior) of the glaucomatous damage [11, 13, 24]. These results suggest that the inferior temporal RNFL is particularly susceptible to the glaucomatous damage and tends to be involved frequently in the initial stage of disease.

Conversely, in advanced glaucoma, the superior GCIPL thickness had the highest correlation with the MD, followed by the superior cpRNFL thickness, whereas the inferior cpRNFL and inferior GCIPL thicknesses had relatively low correlations with the MD (Table 2). In the sectoral analysis in advanced glaucoma, the superior (ST, S, and SN) and some of inferior (IN) GCIPL sectors were also found to have relatively higher correlations with the MD compared to the inferior (I and IT) GCIPL sectors (Figure 3). At advanced stages of glaucoma, it is often difficult for clinicians to detect progression of the disease because extensive structural damage has been occurring. The evaluation of VF parameters constitutes an important part of progression detection. However, due to low reliability and low reproducibility of the VF results in the advanced stage [25], there has been a need for an objective method for assessing glaucomatous damage in the advanced stage. In this regard, the superior GCIPL thickness parameter may represent a complementary tool to the VF assessment for the monitoring of advanced glaucomatous eyes.

Our study had limitations to be acknowledged. First, this study had a cross-sectional study design; to confirm our study finding, longitudinal studies are necessary. Next, the mean age of the advanced glaucoma group is older than that of the other group. As aging could be a potential factor that alters the RNFL and GCIPL, the correlation coefficient and changes of RNFL and GCIPL thicknesses were separately analyzed in early to moderate glaucoma and advanced glaucoma. Finally, potential misclassification of the study groups and inaccurate assessment of disease severity, based on the MD of perimetry, are possible, due to the high test-retest variability of the VF test.

In conclusion, we observed that the distinct patterns of the cpRNFL and GCIPL change in different stages of glaucoma. The findings in this study suggest that the most vulnerable region for initial glaucoma is the inferior RNFL around the optic disc, whereas the most preserved region in advanced glaucoma is the superior macular GCIPL. This information may provide important insights into understanding the natural history of retinal ganglion cell death in glaucoma.

## Supplementary Material

Supplementary Figure 1. The overlay images of fundus photo and RNFL and GCIPL scan. The circumpapillary scan measures on the 6 mm × 6 mm data and the GCA algorithm detects and measures macular GCIPL thickness within an annulus with inner vertical and horizontal diameters of 1 and 1.2 mm, respectively, and outer vertical and horizontal diameters of 4 and 4.8 mm, respectively.

## Figures and Tables

**Table 1:** Demographic and clinical characteristics of the total studied populations.

	Normal controls (*n* = 55)	Early glaucoma (*n* = 190)	Moderate glaucoma (*n* = 58)	Advanced glaucoma (*n* = 48)	*P*	Post hoc
Age (years)	48 ± 12	53 ± 12	53 ± 10	60 ± 13	<0.001	N = E = M < A
Gender (% of female)	41.8	46.3	43.1	62.5	0.399	
Spherical equivalent (diopter)	−1.00 ± 1.62	−1.65 ± 2.31	−1.53 ± 2.61	−2.07 ± 3.18	0.232	
Axial length (mm)	24.0 ± 0.75	24.4 ± 1.2	24.3 ± 1.3	24.3 ± 1.3	0.348	
CCT (*μ*m)	552.0 ± 26.1	538.6 ± 35.9	529.2 ± 30.1	528.9 ± 32.5	0.004	N > E = M = A
MD (dB)	−0.38 ± 1.39	−2.43 ± 1.70	−8.55 ± 1.92	−19.24 ± 5.63	0.000	N > E > M > A
PSD (dB)	1.43 ± 0.29	4.41 ± 2.41	11.13 ± 2.92	11.80 ± 3.02	0.000	N < E < M < A
VFI	100 ± 1	94 ± 5	76 ± 8	45 ± 18	0.000	N > E > M > A
Average cpRNFL thickness (*μ*m)	100.0 ± 7.7	80.3 ± 9.5	70.5 ± 8.9	62.5 ± 8.9	0.000	N > E > M > A
Average macular GCIPL thickness (*μ*m)	85.9 ± 4.3	74.0 ± 6.3	69.1 ± 5.4	59.8 ± 14.0	0.000	N > E > M > A
ONH parameters						
Rim area (mm^2^)	1.31 ± 0.19	0.94 ± 0.20	0.76 ± 0.15	0.63 ± 0.19	0.000	N > E > M > A
Cup area (mm^2^)	0.19 ± 0.16	0.46 ± 0.30	0.51 ± 0.30	0.56 ± 0.31	0.102	N > E = M = A
VCD	0.50 ± 0.11	0.72 ± 0.11	0.77 ± 0.08	0.81 ± 0.07	0.000	N < E < M = A

CCT: central corneal thickness; MD: mean deviation of perimetry; PSD: pattern standard deviation of perimetry; VFI: visual field index; dB: decibels; cpRNFL: circumpapillary retinal nerve fiber layer; GCIPL: ganglion cell-inner plexiform layer; VCD: vertical cup to disc ratio. The severity of the glaucomatous damage was classified into early (MD ≥ −6.00 dB), moderate (−12.00 dB ≤ MD < −6.00 dB), and advanced glaucoma (MD < −12.00 dB). Values are mean ± standard deviation. Comparison among study groups was done by analysis of variance (Tukey multiple comparison).

**Table 2:** Correlation coefficient for MD on Humphrey visual field analysis with circumpapillary RNFL and GCIPL parameters on OCT in different stages of glaucomatous eyes.

	Early to moderate glaucoma^∗^			Advanced glaucoma^∗^		
	*R*	*R* ^2^	*P*	*R*	*R* ^2^	*P*
cpRNFL						
Average	0.477	0.227	<0.001	0.198	0.039	0.177
Superior (quadrant)	0.252	0.063	<0.001	0.452	0.204	0.001
Inferior (quadrant)	0.471	0.221	<0.001	0.192	0.037	0.191
GCIPL thickness						
Average	0.345	0.119	<0.001	0.093	0.009	0.528
Superior	0.159	0.025	0.012	0.495	0.245	<0.001
Inferior	0.397	0.157	<0.001	0.342	0.117	0.020

^∗^The severity of the glaucomatous damage was classified into early (MD ≥ −6.00 dB), moderate, (−12.00 dB ≤ MD < −6.00 dB), and advanced glaucoma (MD < −12.00 dB).
