# Supplementary figures and images for: The Pattern of Retinal Nerve Fiber Layer and Macular Ganglion Cell-Inner Plexiform Layer Thickness Changes in Glaucoma
Source: J Ophthalmol. 2017 Aug 13;2017:6078365. doi: 10.1155/2017/6078365 (PMC5572589; doi:10.1155/2017/6078365)

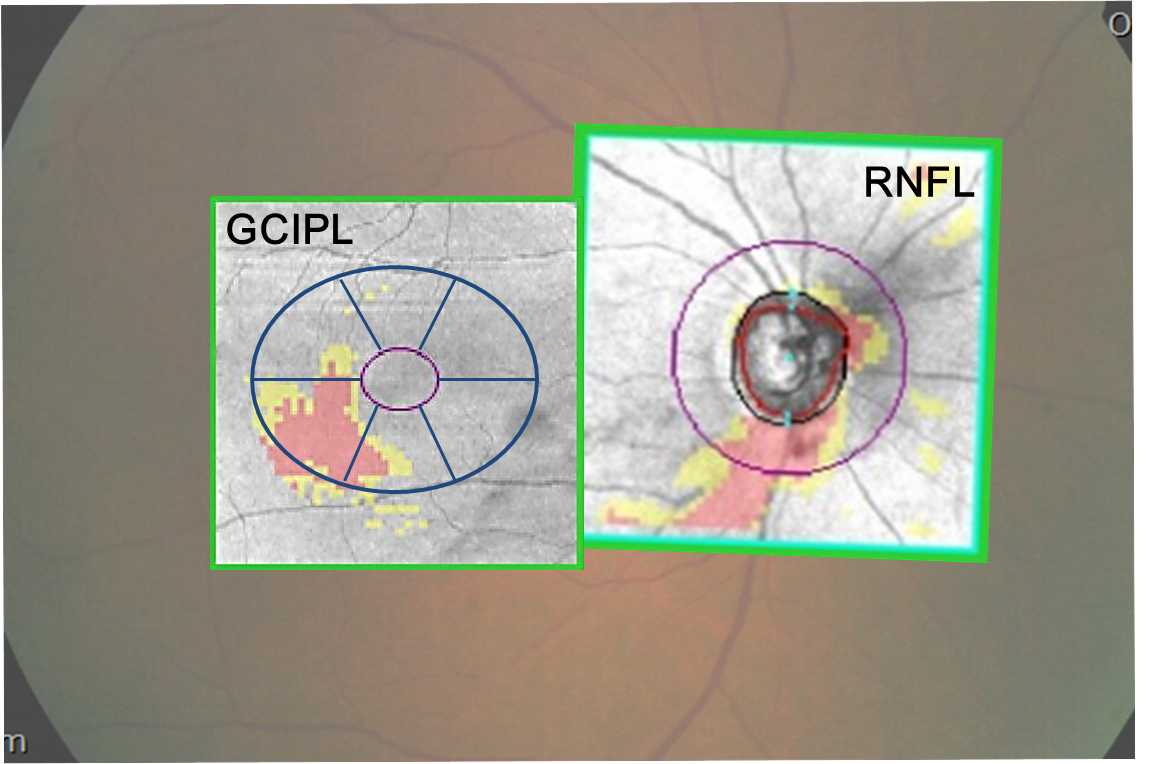

Supplement: Supplementary file 1 — Supplementary Figure 1. The overlay images of fundus photo and RNFL and GCIPL scan. The circumpapillary scan measures on the 6 mm × 6 mm data and the GCA algorithm detects and measures macular GCIPL thickness within an annulus with inner vertical and horizontal diameters of 1 and 1.2 mm, respectively, and outer vertical and horizontal diameters of 4 and 4.8 mm, respectively. [file 6078365.f1.tif]
